# Intranasal exposure to amorphous nanosilica particles could activate intrinsic coagulation cascade and platelets in mice

**DOI:** 10.1186/1743-8977-10-41

**Published:** 2013-08-20

**Authors:** Tokuyuki Yoshida, Yasuo Yoshioka, Saeko Tochigi, Toshiro Hirai, Miyuki Uji, Ko-ichi Ichihashi, Kazuya Nagano, Yasuhiro Abe, Haruhiko Kamada, Shin-ichi Tsunoda, Hiromi Nabeshi, Kazuma Higashisaka, Tomoaki Yoshikawa, Yasuo Tsutsumi

**Affiliations:** 1 Laboratory of Toxicology and Safety Science, Graduate School of Pharmaceutical Sciences, Osaka University, 1-6 Yamadaoka, Suita, Osaka 565-0871, Japan; 2 Laboratory of Biopharmaceutical Research, National Institute of Biomedical Innovation, 7-6-8 Saitoasagi, Ibaraki, Osaka 567-0085, Japan; 3 Cancer Biology Research Center, Sanford Research/USD, 2301 E. 60th Street N, Sioux Falls SD 57104, USA; 4 The Center for Advanced Medical Engineering and Informatics, Osaka University, 1-6 Yamadaoka, Suita, Osaka 565-0871, Japan; 5 Division of Foods, National Institute of Health Sciences, 1-18-1, Kamiyoga, Setagaya-ku, Tokyo 158-8501, Japan

**Keywords:** Nanomaterials, Silica, Platelet, Coagulation

## Abstract

**Background:**

Nanomaterials with particle sizes <100 nm have been already applied in various applications such as cosmetics, medicines, and foods. Therefore, ensuring the safety of nanomaterials is becoming increasingly important. Here we examined the localization and biological responses of intranasally administered amorphous nanosilica particles in mice, focusing on the coagulation system.

**Methods:**

We used nanosilica particles with diameters of 30, 70, or 100 nm (nSP30, nSP70, or nSP100 respectively), and conventional microscale silica particles with diameters of 300 or 1000 nm (mSP300 or mSP1000, respectively). BALB/c mice were intranasally exposed to nSP30, nSP70, nSP100, mSP300, or mSP1000 at concentrations of 500 μg/mouse for 7 days. After 24 hours of last administration, we performed the *in vivo* transmission electron microscopy analysis, hematological examination and coagulation tests.

**Results:**

*In vivo* transmission electron microscopy analysis showed that nanosilica particles with a diameter <100 nm were absorbed through the nasal cavity and were distributed into liver and brain. Hematological examination and coagulation tests showed that platelet counts decreased and that the activated partial thromboplastin time was prolonged in nSP30 or nSP70-treated groups of mice, indicating that nanosilica particles might have activated a coagulation cascade. In addition, in *in vitro* activation tests of human plasma, nanosilica particles had greater potential than did conventional microscale silica particles to activate coagulation factor XII. In nanosilica-particle-treated groups, the levels of soluble CD40 ligand, and von Willebrand factor which are involved in stimulating platelets tended to slightly increase with decreasing particle size.

**Conclusions:**

These results suggest that intranasally administered nanosilica particles with diameters of 30 and 70 nm could induce abnormal activation of the coagulation system through the activation of an intrinsic coagulation cascade. This study provides information to advance the development of safe and effective nanosilica particles.

## Background

Over the past decade, the field of nanotechnology has developed remarkably, and nanomaterials (NMs), which are defined as objects with a diameter less than 100 nm, have been created for various applications. For example, NMs have already been included in many consumer products, such as cosmetics, food, and medicine, to improve their stability and efficacy [1-3]. In particular, amorphous nanosilica particles are one of the most widely applied NMs and are used in cosmetics such as foundation and sunblock, in food additives, and as diluents for medicine [2,4]. As the number of commercial NMs increases, so do opportunities for human exposure to NMs, leading to increasing concern about the safety of NMs [5].

Concerns about the potential health risks of NMs have caused international organizations, such as the World Health Organization and the Organization for Economic Co-operation and Development, to call for an urgent and detailed evaluation of NMs’ safety. To create effective and safer NMs, information about the biodistribution and biological effects of NMs must be acquired. Recent studies have reported that NMs may have unpredicted biological effects that conventional-sized materials do not possess [6,7]. For example, like crocidolite asbestos, carbon nanotubes induce mesothelioma-like lesions in mice [8]. Other reports have shown that exposure to titanium dioxide particles induces inflammatory responses and lung injury in mice [9,10]. In our previous study, we revealed that nanosilica particles could penetrate the skin and enter various tissues [11] and that nanosilica particles can cause pregnancy complications [12], immune-modulating effects [13,14], and consumptive coagulopathy after being absorbed into the whole body of mice [15]. In addition, we showed that nanosilica particles-mediated pregnancy complications and inflammation could be avoided by surface modification of the nanosilica particles with amino or carboxyl groups [12,16], suggesting that modification of the surface of nanosilica particles with amino or carboxyl groups may be effective for the creation of safer nanosilica particles. However, only a few studies have assessed the effects of nanosilica particles by realistic exposure pathways, such as oral or intranasal pathways. In particular, because nanosilica particles are used in the spray products, and inhalation opportunities of nanosilica particles in our life are increasing, such examination of biodistribution and biological responses following nasal exposure routes is urgently needed to advance the use of nanosilica particles in various applications.

Here, we have investigated the *in vivo* localization and biological effects of various sizes of nanosilica particles following intranasal administration in mice. In addition, we examined whether nanosilica particles could influence the coagulation system of mice. We expect that our results will contribute to the creation of safer NMs.

## Results

### Physicochemical examinations of silica particles

In this study, to assess the influence of the size of silica particles on *in vivo* localization or biological effects, we used nanosilica particles with diameters of 30, 70, or 100 nm (nSP30, nSP70, or nSP100, respectively), and conventional microscale silica particles with diameters of 300 or 1000 nm (mSP300 or mSP1000, respectively). In a previous study, we confirmed that the mean secondary particle diameters of each of these types of silica particles are 39, 76, 106, 264, and 1136 nm (for nSP30, nSP70, nSP100, mSP300, and mSP1000, respectively) by dynamic laser scattering analysis [11,12,14], and all the particles were confirmed to be well-dispersed smooth-surfaced spheres by transmission electron microscopy. These results indicate that the silica particles used in this study would remain stable and well-dispersed in solution, without aggregating.

### *In vivo* localization of silica particles

**Figure 1:**
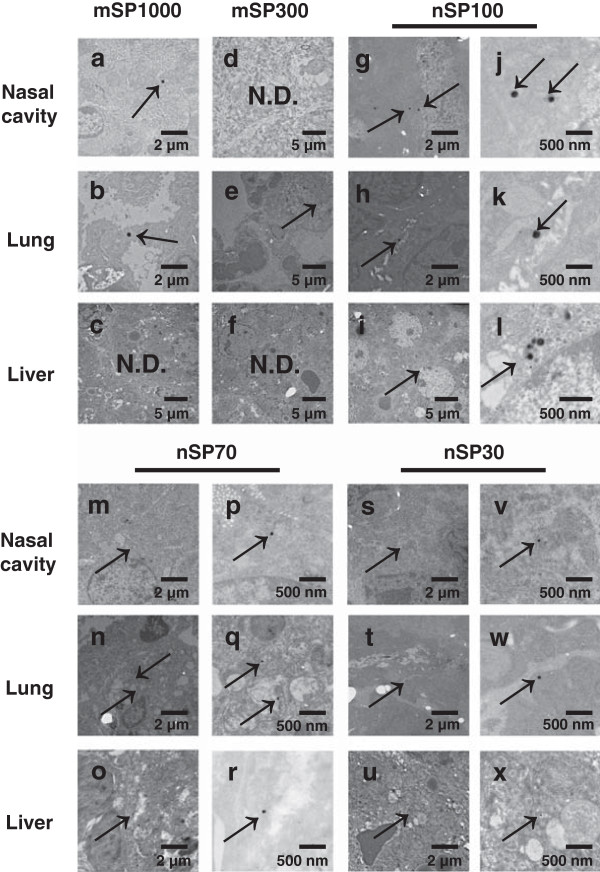
**Biodistribution of silica particles.** BALB/c mice were intranasally exposed to nSP30, nSP70, mSP300, nSP100, or mSP1000 at a concentration of 500 μg/mouse for 7 days. Twenty-four hours after the final administration, the nasal cavity **(a**,**d**,**g**,**j**,**m**,**p**,**s**,**v)**, lung **(b**,**e**,**h**,**k**,**n**,**q**,**t**,**w)**, and liver **(c**,**f**,**i**,**l**,**o**,**r**,**u**,**x)** were evaluated by transmission electron microscopy. Arrows point to silica particles. N.D.: not detected.

First, we qualitatively examined the *in vivo* localization of silica particles after intranasal administration (Figure 1). BALB/c mice were intranasally exposed to nSP30, nSP70, nSP100, mSP300, or mSP1000 at concentrations of 500 μg/mouse for 7 days. Transmission electron microscopy analysis revealed that mSP1000 were located in mucosal epithelial cells of the nasal cavity (Figure 1a), and mSP300 and mSP1000 were located in type II alveolar epithelial cells of the lung (Figure 1b,e), although they were not detected in the liver (Figure 1c,f). On the other hand, nSP30, nSP70, and nSP100 were located not only in the nasal cavity (Figure 1g,j,m,p,s,v) and lung (Figure 1h,k,n,q,t,w) but also in hepatocytes in the liver (Figure 1i,l,o,r,u,x). These results suggested that nanosilica particles were absorbed through the nasal cavity and distributed into some tissues in the body. Therefore, to thoroughly evaluate the safety of these NMs, the biological effects of the intranasally administered silica particles might need to be evaluated for all tissues in the mouse body.

### Biological effects induced by silica particles in tissue

**Figure 2:**
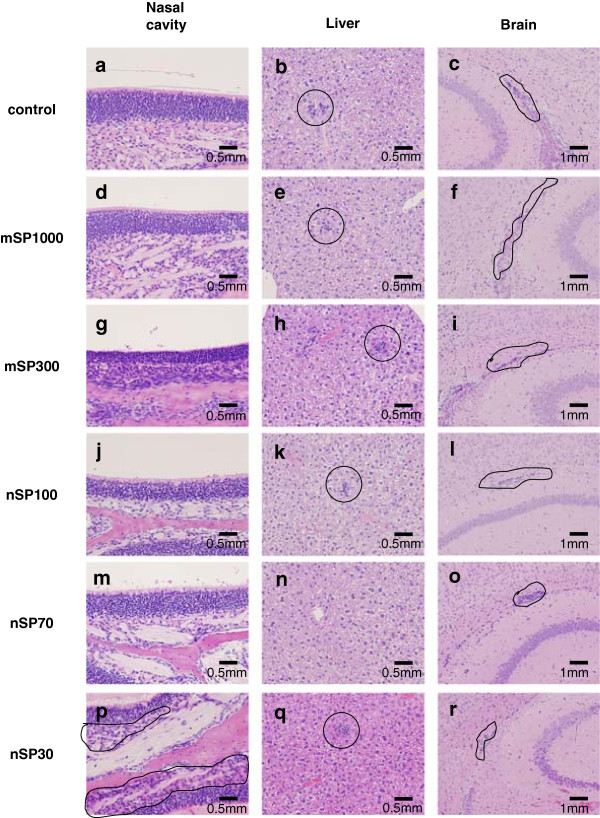
**Histopathological analysis.** BALB/c mice were intranasally exposed to nSP30, nSP70, mSP300, nSP100, or mSP1000 at a concentration of 500 μg/mouse for 7 days. Twenty-four hours after the final administration, nasal cavity **(a**,**d**,**g**,**j**,**m**,**p)**, liver **(b**,**e**,**h**,**k**,**n**,**q)** and brain **(c**,**f**,**i**,**l**,**o**,**r)** tissue samples were stained with hematoxylin–eosin. In images, solid line delineates cell aggregation. The histopathological grades of these samples are summarized in Table 1.

**Figure 3:**
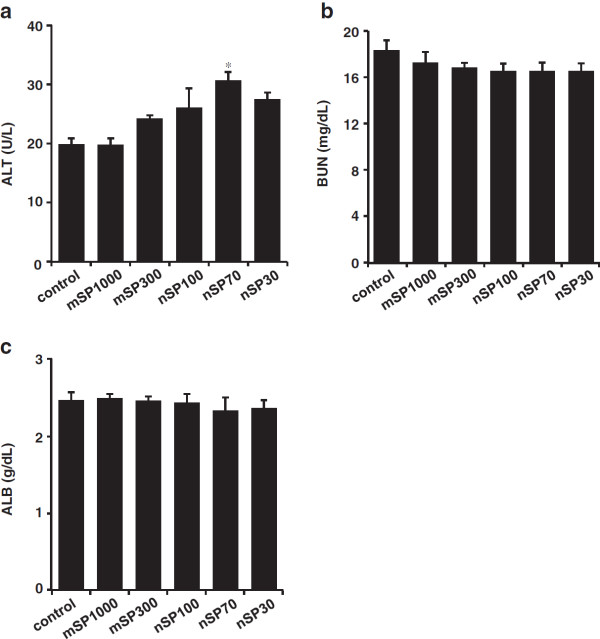
**Biochemical analysis.** BALB/c mice were intranasally exposed to nSP30, nSP70, mSP300, nSP100, or mSP1000 at a concentration of 500 μg/mouse for 7 days. Twenty-four hours after the final administration, plasma was collected. **(a)** alanine aminotransferase (ALT), **(b)** blood urea nitrogen (BUN), and **(c)** albumin (ALB) were analyzed. Results are expressed as mean ± S.E. (*n* = 4–5). *Represents significant difference from the control (*p* < 0.05).

To evaluate the effects of silica particles on the tissue in which they were present after intranasal administration, we observed the nasal cavity, brain, and liver of each mouse by hematoxylin–eosin staining (Figure 2). For all the groups of mice, although very slight inflammatory cell aggregation was observed in the nasal cavity, brain, and liver, these pathological findings were within normal ranges (Figure 2 and Table 1). Next, we measured the liver damage markers alanine aminotransferase (ALT) and albumin (ALB), as well as kidney damage marker blood urea nitrogen (BUN), in the tissues (Figure 3). Although the level of ALT in plasma increased slightly in nanosilica-particle-treated groups compared to the control group, the change in the ALT value was within a normal, healthy range (<43 U/L) among all the groups (Figure 3a). BUN and ALB levels did not change significantly for any of the groups (Figure 3b,c). These results suggest that the intranasally administered nanosilica particles did not induce abnormal changes in some tissues.

**Table 1:** Histopathological grades of nasal cavity, brain, and liver tissue samples collected from three different BALB/c mice exposed to nSP30, nSP70, mSP300, nSP100, or mSP1000 via intranasal administration

**Findings**	**Control**	**mSP1000**	**mSP300**	**nSP100**	**nSP70**	**nSP30**
**Nasal cavity**						
Cell aggregation	0 0 0	0 0 0	0 0 0	0 0 0	0 0 0	1 1 1
**Brain**						
Microglial aggregation	1 1 0	1 1 0	1 2 1	1 0 0	2 1 0	1 1 1
**Liver**						
Cell aggregation	1 2 0	1 2 1	1 1 0	1 1 1	0 0 0	0 1 1
Rarefaction	3 3 2	3 3 2	3 3 3	3 3 3	3 2 2	3 3 2

Grade; 0: none, 1: very slight, 2: mild, 3: moderate, 4: advanced.

**Figure 4:**
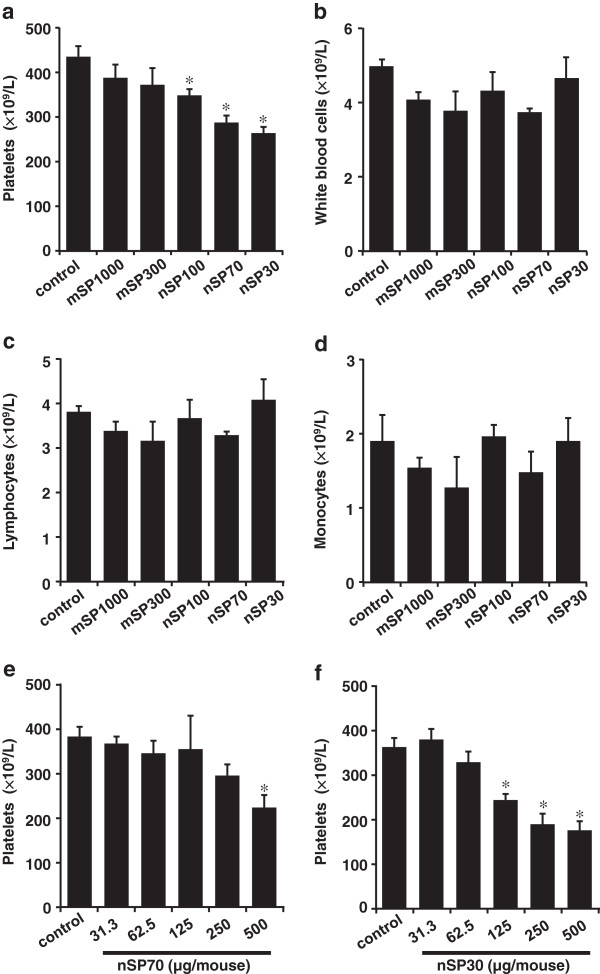
**Hematological analysis.** BALB/c mice were intranasally exposed to nSP30, nSP70, mSP300, nSP100, or mSP1000 at a concentration of 500 μg/mouse in **(a**-**d)**, and 31.3-500 μg/mouse in **(e**,**f)** for 7 days. Twenty-four hours after the final administration, **(a**,**e**,**f)** platelets, **(b)** white blood cells, **(c)** lymphocytes, and **(d)** monocytes were analyzed. Results are expressed as mean ± S.E. (*n* = 5). *Represents significant difference from the control (*p* < 0.05).

Next, we performed hematological examination. As the size of the nanosilica particles decreased, so did the platelet counts in the silica-particle-treated groups, although the counts of other blood components (white blood cells, lymphocytes, and monocytes) remained unchanged in all groups (Figure 4a–d). The decrease in platelets was confirmed to occur in a dose-dependent manner in the nSP70- and nSP30-treated groups (Figure 4e,f). The platelet counts for nSP70 at concentrations below 250 μg/mouse and for nSP30 at concentrations below 62.5 μg/mouse were equal to those of the control group (Figure 4e,f). Thus, these findings suggest that intranasally administered nanosilica particles may decrease platelet counts.

### Activation of coagulation system induced by silica particles

**Figure 5:**
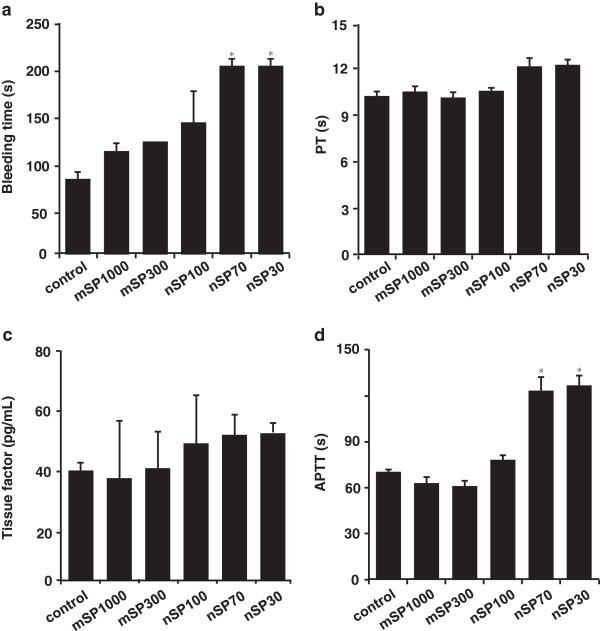
**Examination of coagulation cascade.** BALB/c mice were intranasally exposed to nSP30, nSP70, mSP300, nSP100, or mSP1000 at a concentration of 500 μg/mouse for 7 days. Twenty-four hours after the final administration, **(a)** bleeding time (evaluated by Duke’s method), **(b)** prothrombin time (PT) and **(d)** activated partial thromboplastin time (APTT) were evaluated. **(c)** The level of tissue factor (TF) in plasma was determined using ELISA. PT and APTT in collected plasma were determined at 37°C in the Clotek dry-block heating system with PT and APTT reagents. Results are expressed as mean ± S.E. (*n* = 4–5). *Represents significant difference from the control (*p* < 0.01).

Previously, we revealed that a drastic decrease in platelets after systemic exposure to nSP70 could result from consumptive coagulopathy [15]. Therefore, we speculated that intranasally administered nanosilica particles might also induce a coagulation cascade. To evaluate the effect of nanosilica particles on the coagulation cascade, we measured the bleeding time of whole blood from each silica-particle-treated mouse by Duke’s method (Figure 5a). Bleeding time was prolonged in the nSP30- and nSP70-treated groups compared to the control group, although the bleeding times of the nSP100-, mSP300-, and mSP1000-treated groups did not change (Figure 5a). These results suggest that intranasal exposure to nanosilica particles could induce abnormal activation of the coagulation system and thus leads to the consumptive coagulopathy.

Next, we examined the mechanism of abnormal activation of the coagulation system induced by nanosilica particles after intranasal administration. The blood coagulation system can be initiated by two pathways: an extrinsic cascade pathway, which is triggered by the release of tissue factor (TF) from the site of injury, and an intrinsic cascade pathway, which is triggered by activation of coagulant factor contacted with a negatively charged substance or accumulation of activated platelets to the collagen layer under the vascular endothelium [17]. We examined which cascade pathway was involved in the abnormal activation of the coagulation cascade by nanosilica particles after intranasal administration. The levels of prothrombin time (PT) (Figure 5b) and TF (Figure 5c), parameters for the activation of an extrinsic coagulation pathway, and activated partial thromboplastin time (APTT) (Figure 5d), a parameter for the activation of an intrinsic coagulation pathway, were measured in the plasma of each silica-particle-treated mouse. The levels of PT and TF did not vary compared to those observed for the control group (Figure 5b,c). In contrast, APTT in plasma from the nSP30- or nSP70-treated group was remarkably prolonged compared to that of the control group, although no change in APTT was observed for the nSP100-, mSP300-, or mSP1000-treated group (Figure 5d). These results suggest that the activation of an intrinsic cascade pathway induced by nanosilica particles could result in abnormal activation of the coagulation system.

**Figure 6:**
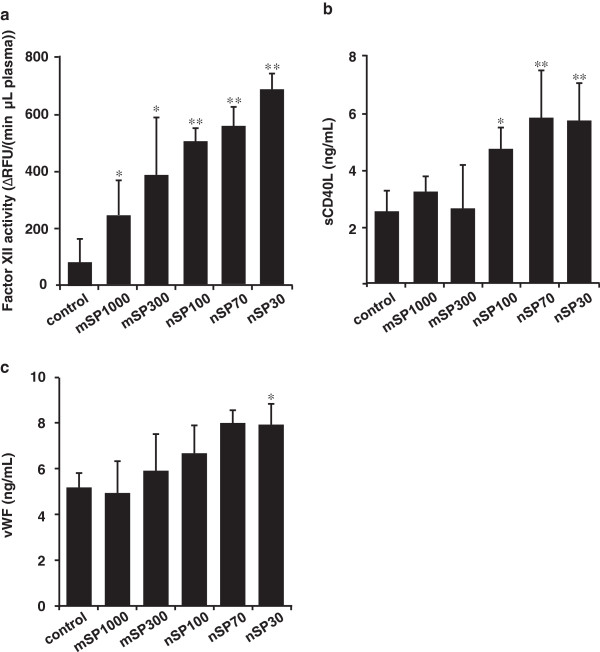
**Examination of intrinsic cascade pathways. (a)***In vitro* changes in blood coagulation factor XII activity in human plasma by silica particles of various sizes. The initial rate of reaction of coagulation factor XII was obtained from human plasma, which was obtained from a control sample of human plasma, which did not contain any silica particles, and was measured by fluorescence intensity. Results are expressed as mean ± S.D. (*n* = 5). ** and * represent significant differences from the control (**p < 0.01, *p < 0.05). RFU: relative fluorescent unit. **(b)** The level of soluble CD40 ligand (sCD40L) and **(c)** the level of von Willebrand factor (vWF) in mouse plasma. BALB/c mice were intranasally exposed to nSP30, nSP70, mSP300, nSP100 or mSP1000 at a concentration of 500 μg/mouse for 7 days. Twenty-four hours after the final administration, the levels of sCD40L and vWF in the mouse plasma were determined using ELISA. Results are expressed as mean ± S.E. (*n* = 4–5). ** and * represent significant differences from the control (**p < 0.01, *p < 0.05).

Generally, the activation of an intrinsic cascade pathway is initiated by coagulation factor XII when it comes into contact with hydrophilic activating particles (such as fully water-wettable glass) [18]. Platelet activation is also involved in the activation of intrinsic cascade pathways [19]. We thus performed *in vitro* activation tests of coagulation factor XII using human plasma to confirm the presence of an intrinsic cascade pathway. One of our overall research goals is to contribute to the development of NMs that will be effective as well as safe for human exposure. To conduct a preliminary evaluation of the effects of nanosilica particles on humans, we used human plasma in the experiment, rather than mouse plasma. The *in vitro* activation tests showed that all sizes of the silica particles had the potential to activate coagulation factor XII, with activation apparently increasing as the size of the particles decreased (Figure 6a). In addition, to evaluate the activation of platelets, we measured the level of soluble CD40 ligand (sCD40L) and von Willebrand factor (vWF), which are involved in stimulating platelets [20,21], in the plasma of each silica-particle-treated mouse. In nanosilica-particle-treated groups, the levels of sCD40L and vWF tended to slightly increase with decreasing particle size (Figure 6b,c). These results suggest that the activation of an intrinsic coagulation pathway by nanosilica particles after intranasal administration was promoted by the activation of coagulation factor XII and platelets.

## Discussion

Merget *et al*. suggested that the inhalation of amorphous silica particles in the workplace could induce silicosis [22]. In addition, nanosilica particles have been explored for medical applications, such as cancer therapeutics or drug-delivery agents *via* intranasal administration [23,24]. However, there is not enough information about the biological effects of nanosilica particles after intranasal exposure for them to be used safely. In this study, we focused on intranasal exposure of nanosilica particles and determined the localization and biological effects of nanosilica particles after intranasal administration.

Warheit *et al.* and Lee *et al.* reported that the no-observable-effect level (NOEL) of colloidal silica particles is 489 μg/lung (equivalent to an inhalation exposure of 10 mg/m^3^) [25,26]. Accordingly, we designed our experiments such that mice were intranasally exposed to various sizes of silica particles at 500 μg/mouse for 7 days, a level close to the NOEL for inhalation exposure. The dose in our study is important from the viewpoint of establishing an upper threshold for the amount of NMs that can safely be administered intranasally. Although we still need to accumulate much more information about the biological effects of nanosilica particles using intranasal administration, at realistic exposure levels, we expect that our present study will contribute to the safety assessment of NMs.

We found that nSP30, nSP70, and nSP100 were located not only in the nasal cavity and lung but also in the liver (Figure 1). In our previous study, we showed that nSP70, mSP300, and mSP1000 localized in the liver after entering the bloodstream [11]. When we hypothesize how the nanosilica particles (smaller than 100 nm) enter the liver after intranasal administration, it is important to discuss how nanosilica particles enter the bloodstream through the nasal cavity or lung. We hypothesized that the nanosilica particles were absorbed through transcytosis, or uptake by microfold cells (M cells) in bronchus-associated lymphoid tissues and nasal-associated lymphoid tissues. Other reports have suggested that NMs open the tight junction, which plays an important role in maintaining the epithelial barrier [27,28]. Thus, to investigate the pathway by which nanosilica particles enter the body, we need to evaluate the effects of nanosilica particles on M cells or epithelial cell barriers *in vitro.* In this study, we examined only the nasal cavity, lung, and liver, so we cannot comment as to whether nanosilica particles are localized in other tissues. However, other groups have reported that titanium dioxide nanoparticles with a diameter of 80 nm were localized in the brain after intranasal administration [29,30]. Furthermore, Liu *et al.* showed that copper nanoparticles with a diameter of 23.5 nm enter the olfactory bulb in the brain [31]. Therefore, in our study, it is possible that nanosilica particles with diameters of 30 or 70 nm may have been localized in the brain. In this analysis, the particles detected in tissues after intranasal administration of mSP300 and mSP1000 were smaller than the average diameter of the respective administered particles (Figure 1a,b,e). We consider the possibility of degradation of mSP300 and mSP1000 in the body, because some previous *in vitro* studies have suggested that silica particles could be degraded in humans and animals after absorption [32-34]. For example, Kim *et al.* showed that approximately 50% to 80% of a sample of microsilica particles was dissolved within 36 h in a solution of phosphate-buffered saline with 10% bovine serum, which is a simulated body fluid [32]. In addition, a recent study suggested the possibility of silica particle degradation at a cellular level. Zhai *et al.* showed that hollow mesoporous nanosilica particles degraded when injected into human umbilical vein endothelial cells [33]. On the basis of these reports’ findings, silica particles localized in biological bodies might be degraded within 7 days by means of interaction with biological fluid or by uptake into epithelial cells in the nasal cavity or lungs. On the other hand, because transmission electron microscopy analysis is only a qualitative method, we need to quantitatively analyze the silica particles after intranasal exposure to obtain more detailed information about their biodistribution. Inductively coupled plasma–optical emission spectrometry (ICP-OES) is reported to be a suitable means for quantitatively measuring silica. Using ICP-OES, we initially attempted to quantify the absorption of nSP30 and nSP70 in the liver after intranasal exposure for 7 days. However, we did not detect the particles in biological tissue using ICP-OES (data not shown; the detection limit of our protocol was 50 μg/g). In our study, nanosilica particles were not localized in the liver at levels sufficient for measurement by ICP-OES. To quantitatively analyze the nanosilica particles and clarify their absorption, distribution, metabolism, and excretion mechanisms, a method with greater sensitivity must be developed.

Previously, we found that nanosilica particles could accumulate in the liver and induce severe liver damage after intravenous administration [11,15]. The level of nanosilica particles accumulated in the liver after intranasal administration would be lower than that observed after intravenous administration, and thus a nanosilica-particle-mediated increase of ALT levels or abnormal findings in pathological examination would have been reduced in the present study. We must measure the level of nanosilica particles in the liver quantitatively to confirm this speculation; overall, our present findings suggest that we need to more precisely evaluate the biological effects of intranasally administered NMs on all tissues in the body, including the liver and brain.

Intranasally administered nanosilica particles might have induced abnormal activation of the intrinsic coagulation cascade (Figures 4 and 5). To explain the decrease of platelets observed in the nanosilica-particle-treated groups, the nanosilica particles might directly activate the platelets and promote the coagulation cascade, resulting in consumption of platelets and consequently prolonged bleeding times. Other groups have shown that some NMs, such as single-walled carbon nanotubes and rutile titanium dioxide nanorods, could activate platelets and induce abnormal activation of the coagulation system [35-38]. Therefore, in our study the platelets might have been activated by the nanosilica particles and then subsequently consumed as they formed blood clots, thus decreasing the number of platelets and consequently prolonging bleeding time. Furthermore, the platelet counts in the nSP70-treated group at concentrations <250 μg/mouse and in the nSP30-treated group at concentrations <62.5 μg/mouse were equal to the count of the control group. Thus, this finding could provide useful information for setting the no-observable-adverse-effect level for intranasally administered nanosilica particles. Our present results indicate that the abnormal activation of a coagulation cascade by nanosilica particles after intranasal administration was promoted by activation of an intrinsic cascade pathway (Figure 5). However, our previous study showed that intravenously administered nSP70 could induce the release of TF, which is a known marker of activation of an extrinsic cascade pathway [15]. We speculate that the level of intranasally administered nanosilica particles in the bloodstream was lower than that of intravenously administered nanosilica particles, and thus a drastic release of TF was not detected in this study.

Contact activation of coagulation factor XII is one of the major factors of blood coagulation [39,40], and, as mentioned earlier, coagulation factor XII is activated when it comes into contact with hydrophilic activating particles (such as fully water-wettable glass) [18]. Since the number of silica particles per unit weight increases as the particle size decreases (the particle numbers of the silica particles were 3.5 × 10^13^, 2.8 × 10^12^, 9.5 × 10^11^, 3.5 × 10^10^, and 9.5 × 10^8^ particles/mg for nSP30, nSP70, nSP100, mSP300, and mSP1000, respectively), the number of opportunities for contact between the nanosilica particles and coagulation factor XII would have increased with decreasing particle size, thus ultimately leading to the activation of coagulation factor XII. In addition, we need to take into account not only the number of silica particles but also the surface area. The intrinsic cascade pathway involves various factors, such as factor XI and prekallikrein. Therefore, to reveal the mechanism of abnormal activation of the coagulation cascade by nanosilica particles, we need to examine the effects of nanosilica particles on other factors in intrinsic cascade pathways. Increases in the levels of sCD40L and vWF were observed in plasma from the nSP30- and nSP70-treated groups (Figure 6), meaning that nanosilica particles absorbed into the bloodstream induced activation of platelets, which are involved in the activation of coagulation pathways. Although we need to evaluate in greater detail the effects of nanosilica particles on activation or aggregation of platelets, our results and these previous reports suggest that nanosilica particles would induce platelet activation, resulting in activation of an intrinsic cascade pathway. Tavano *et al.* suggested that synthetic amorphous silica (SAS), which is similar to our silica nanoparticles, and organically modified silica (ORMOSIL) nanoparticles induce significant abnormal activation of the coagulation system *via* a different mechanism in *in vitro* studies [41]. More specifically, SAS nanoparticles activate contact coagulation (factor XII dependent) but not TF transcription in monocytes. In contrast, ORMOSIL nanoparticles induce TF-dependent coagulation more efficiently than SAS nanoparticles. The group’s report indicated that the activation of an intrinsic cascade pathway is the main mechanism of amorphous silica nanoparticle–mediated procoagulant activity, thus supporting our study’s conclusions and reiterating the importance of examining the effects of silica nanoparticles on intrinsic coagulation.

In summary, we revealed that intranasally administered nanosilica particles have the potential to induce abnormal activation of a coagulation cascade in mice. Recently, nanosilica particles have been used in food additives and cosmetics, and thus opportunities for such particles to be inhaled by workers during manufacturing are increasing [4,42]. Furthermore, nanosilica particles are being explored as cancer therapy and drug-delivery agents, and might be administered intranasally in such applications as well [23,24]. Therefore, the localization and biological effects of intranasally administered nanosilica particles must be elucidated. We expect that further studies of the relationship between localization and biological effects will provide useful information for the development of safer, effective NMs.

## Conclusions

We have shown that nanosilica particles with diameters of 30, 70, and 100 nm intranasally administered to mice were absorbed into the bloodstream and distributed into certain organs, such as the liver. The obtained results suggest that the activation of an intrinsic cascade pathway induced by nanosilica particles with diameters of 30 and 70 nm, both of which activated coagulation factor XII and platelets, could result in abnormal activation of the coagulation system. We expect that the findings of this study will contribute to the ongoing development of NMs that are safe for use in humans and animals.

## Methods

### Silica particles

Amorphous nanosilica particles with diameters of 30, 70, and 100 nm, as well as microscale silica particles with diameters of 300 and 1000 nm (Micromod Partikeltechnologie, Rostock/Warnemünde, Germany, designated nSP30, nSP70, nSP100, mSP300, and mSP1000, respectively) were used in this study. The particle numbers of the silica particles were 3.5 × 10^13^, 2.8 × 10^12^, 9.5 × 10^11^, 3.5 × 10^10^, and 9.5 × 10^8^ particles/mg for nSP30, nSP70, nSP100, mSP300, and mSP1000, respectively. Each type of silica particles was sonicated for 5 min and vortexed for 1 min before use.

### Animals

BALB/c mice (female, 6–8 weeks) were purchased from Japan SLC, Inc. (Shizuoka, Japan). Mice were housed in a ventilated animal room maintained at 20 ± 2°C with a 12-h light/12-h dark cycle. Mice had free access to water and alfalfa-free forage (FR-2, Funabashi Farm, Funabashi, Japan). All of the animal experimental procedures in this study were performed in accordance with the National Institute of Biomedical Innovation and Osaka University Guidelines for the Welfare of Animals.

### Transmission electron microscopy analysis

Five BALB/c mice were intranasally exposed to a 20 μL aliquot (10 μL per nostril) of nSP30, nSP70, nSP100, mSP300, or mSP1000 at a concentration of 500 μg/mouse for 7 days. Twenty-four hours after the final intranasal administration, the nasal cavity, lung, and liver from two mice were excised and fixed in 2.5% glutaraldehyde for 2 h. Then, small pieces of tissue sample were washed with phosphate buffer 3 times and postfixed in sodium cacodylate-buffered 1.5% osmium tetroxide for 60 min at 4°C, block-stained in 0.5% uranyl acetate, dehydrated by dipping each sample through a series of ethanol solutions containing increasing concentration of ethanol, and embedded in Epon resin (TAAB). Ultrathin sections were stained with uranyl acetate and lead citrate. The stained samples were subsequently observed under an electron microscope (H-7650, Hitachi, Tokyo, Japan).

### Histopathological examination

Twenty-four hours after the final intranasal administration of each type of silica particles, the nasal cavity, lung, and liver from three mice were excised and fixed immediately in 4% paraformaldehyde. These tissues were embedded in paraffin blocks and then sliced, and the slices were placed on glass slides. After hematoxylin–eosin staining, the slides were observed, and cell aggregation in the nasal cavity, microglial aggregation in the brain, and cell aggregation and rarefaction in the liver were classified into one of five grades (0: none, 1: very slight, 2: mild, 3: moderate, 4: advanced).

### Blood biomarker assay

Twenty-four hours after the final intranasal administration of each type of silica particles, blood samples were collected from the heart using plastic syringes (Terumo, Tokyo, Japan) containing 5 IU/mL heparin sodium. Plasma was harvested by centrifuging the blood at 1750 × *g* for 15 min. The levels of ALT, ALB and BUN were determined in the plasma using a biochemical auto-analyzer (Fuji dri-Chem 7000, Fujifilm, Tokyo, Japan).

### Hematology analysis

Twenty-four hours after the final intranasal administration of each type of amorphous silica particles, blood samples were collected from the heart using plastic syringes (Terumo) containing 0.1 mM EDTA. Whole blood samples were analyzed with a VetScan HMII Hematology System (Abaxis, Sunnyvale, CA, USA) to determine the number of white blood cells, lymphocytes, monocytes, and platelets.

### Measurement of bleeding time and coagulation tests

Twenty-four hours after the final intranasal administration of each type of silica particles, the bleeding time for each mouse was measured by Duke’s method [43]. Briefly, an ear of each mouse was cut with a knife, and the blood generated at the site of the cut was absorbed with filter paper every 30 seconds until bleeding ceased. To examine the coagulation tests in each mouse, a blood sample was collected from the heart of each mouse subjected to bleeding-time tests using plastic syringes (Terumo) containing 1:9 (v/v) of 3.8% sodium citrate. Plasma was harvested by centrifuging the blood at 1750 × *g* for 15 min. APTT and PT levels were determined at 37°C in a Clotek dry-block bath system (Hyland Division, Travenol Laboratories, Inc. Costa Mesa, CA, USA) with APTT and PT reagents (Sysmex, Kobe, Japan), respectively.

### *In vitro* activation tests of coagulation factor XII

One hundred microliters of human health plasma (Scipac, Kent, UK) and 100 μL of various sized silica particles (3.13 mg/mL) were mixed for 1 min at room temperature with an enzyme reaction solution (50 mM Tris–HCl, 0.15 M NaCl, 1 mM CaCl_2_ and 0.1 mg/mL bovine serum albumin, pH 8.0) containing 2 mM *t*-butyloxycarbonyl-l-glutaminyl-glycyl-l-arginine-4-methyl-courmaryl-7-amide (Peptide Institute, Inc. Ibaraki, Japan) in dimethyl sulfoxide. The initial rate of reaction was calculated by measuring the fluorescence intensity (380 nm excitation, 440 nm emission) every 5 min. The initial rate is given by Initial rate = (relative fluorescent unit of each measurement time – blank) / measurement time.

### Detection of TF, sCD40L, and vWF levels in plasma

TF, sCD40L, and vWF levels in plasma were determined using enzyme-linked immunosorbent assay (ELISA) kits (TF: Mouse Tissue Factor ELISA kit (Cusabio, Newark, DE, USA); sCD40L: Mouse sCD40L ELISA (eBioscience, San Diego, CA, USA); vWF: Mouse von Willebrand Factor ELISA kit (Cusabio, Newark, DE, USA)).

### Statistical analysis

Differences among each group were compared by using Williams’s or Dunnett’s method after analysis of variance (ANOVA).

## Competing interests

The authors declare that they have no competing interests.

## Authors’ contributions

TY and YY designed the study. TY, ST, TH, MU, and KI performed the experiments. TY and YY collected and analyzed the data. TY and YY wrote the manuscript. KN, YA, HK, ST, HN, KH, and TY provided technical support and conceptual advice. YT supervised the project. All authors discussed the results and commented on the manuscript. All authors read and approved the final manuscript.
